# Demographic history and gene flow in the peatmosses *Sphagnum recurvum* and *Sphagnum flexuosum* (Bryophyta: Sphagnaceae)

**DOI:** 10.1002/ece3.9489

**Published:** 2022-11-16

**Authors:** Karn Imwattana, Blanka Aguero, Aaron Duffy, A. Jonathan Shaw

**Affiliations:** ^1^ Department of Biology & L. E. Anderson Bryophyte Herbarium Duke University Durham North Carolina USA

**Keywords:** demographic history, effective population size, gene flow, genetic diversity, glaciation, *sphagnum*

## Abstract

Population size changes and gene flow are processes that can have significant impacts on evolution. The aim of this study was to investigate the relationship of geography to patterns of gene flow and population size changes in a pair of closely related *Sphagnum* (peatmoss) species: *S. recurvum* and *S. flexuosum*. Both species occur in eastern North America, and *S. flexuosum* also occurs in Europe. Genetic data from restriction‐site‐associated DNA sequencing (RAD‐seq) were used in this study. Analyses of gene flow were accomplished using coalescent simulations of site frequency spectra (SFSs). Signatures of gene flow were confirmed by *f*_*4*_ statistics. For *S. flexuosum*, genetic diversity of plants in glaciated areas appeared to be lower than that in unglaciated areas, suggesting that glaciation can have an impact on effective population sizes. There is asymmetric gene flow from eastern North America to Europe, suggesting that Europe might have been colonized by plants from eastern North America after the last glacial maximum. The rate of gene flow between *S. flexuosum* and *S. recurvum* is lower than that between geographically disjunct *S. flexuosum* populations. The rate of gene flow between species is higher among sympatric plants of the two species than between currently allopatric *S. flexuosum* populations. There was also gene flow from *S. recurvum* to the ancestor *S. flexuosum* on both continents which occurred through secondary contact. These results illustrate a complex history of interspecific gene flow between *S. flexuosum* and *S. recurvum*, which occurred in at least two phases: between ancestral populations after secondary contact and between currently sympatric plants.

## Graphical abstract

This study has shown that there are multiple phases of gene flow between closely related peatmosses. Most of the gene flow occured in the past via secondary contact, the lesser extent of gene flow is currently ongoing between only sympatric populations.

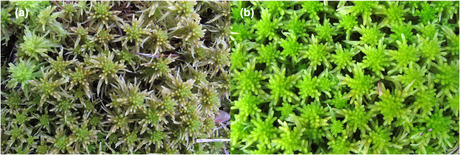

## 1 INTRODUCTION

Inferring patterns of demography and gene flow among diverging populations is crucial to understanding speciation processes (Edelman & Mallet, 2021; Ellstrand, 2014; Nielsen et al., 2009). Gene flow, the movement of genetic material between individuals from differentiated populations, can act as a homogenizing force among partially divergent species. In some instances, however, gene flow can augment genetic diversity within populations and provide potentially adaptive alleles or even promote the speciation process (Abbott et al., 2013; Morjan & Rieseberg, 2004; Richards & Martin, 2017; Slatkin, 1987; Suarez‐Gonzalez et al., 2018). Rates of gene flow can obviously be affected by physical distances; proximate individuals are more likely to exchange genetic material than more distant ones. Gene flow can occur between populations within the same species (intraspecific gene flow) and between populations of different species (interspecific gene flow). Since individuals from different species usually have some degree of reproductive isolation, interspecific gene flow in general should occur at lower levels than intraspecific gene flow (Edelman & Mallet, 2021; Ellstrand, 2014). Changes in population size through time can also influence the genetic makeup of populations. For example, a population expansion can create an excess of rare alleles that can mimic signatures of selection. Population sizes can be influenced by changes in environmental conditions that cause populations to contract or expand (Nielsen et al., 2009). Major environmental changes such as glaciation can have profound effects on population sizes (Abbott & Brochmann, 2003). Founder events following dispersal or range expansions can also significantly impact the genetic makeup of populations (Hewitt, 1996).

Developments in sequencing technologies have made genome‐scale data in non‐model organisms much easier to acquire. Such large datasets allow for the analyses of complex evolutionary models (Ekblom & Galindo, 2011). Statistical methods have been developed to compare demographic models that include different effective population sizes through time and variable gene flow histories (Beichman et al., 2018; Excoffier et al., 2013). This can be instrumental in understanding the speciation process of closely related species, which involves a complex interaction of population divergence, population size changes, and gene flow.

Peatmosses (*Sphagnum* spp.) are semiaquatic to terrestrial plants that grow in bogs, fens, forests, and seepages (Rydin et al., 2013). *Sphagnum* is of unparalleled ecological importance because some 25%–30% of the entire terrestrial pool of carbon is estimated to be bound up in partially decomposed peat within *Sphagnum*‐dominated peatlands (Gorham, 1991; Yu, 2011). Thus, understanding evolutionary and ecological processes in *Sphagnum* has profound implications for biogeochemistry and the control of global climate (Weston et al., 2018).

There are around 300–500 species of *Sphagnum* worldwide, and although the *Sphagnum* clade is hundreds of millions of years old, most extant species seem to have emerged through relatively recent diversification during the last 10–15 million years (Shaw et al., 2010). *Sphagnum* is capable of long‐distance dispersal via either spores or vegetative fragments (Sundberg, 2013). Many species have intercontinental ranges, with some degree of population structure across their geographic ranges (Kyrkjeeide et al., 2016). Long‐distance dispersal allows populations in different geographic regions, even between continents, to remain connected by gene flow (Shaw et al., 2015; Stenøien et al., 2011). Multiple species of *Sphagnum* often occupy the same habitat, usually by specializing in different microhabitats. In fact, many sites have ten or more sympatric species. Different *Sphagnum* species in the same habitat can hybridize, at least occasionally (Cronberg, 1998; Cronberg & Natcheva, 2002). In addition to hybridization occurring in current populations, recent analyses using genomic data showed signatures of ancient introgressions between *Sphagnum* species (Meleshko et al., 2021). Many *Sphagnum* species occur in northern areas that were covered by ice during the last glacial maximum (LGM) and have experienced significant shifts in geographical range during the recent past (Abbott & Brochmann, 2003; Gignac et al., 2000). These attributes, interspecific gene flow, recent range changes, and the potential for long distance dispersal can make the demographic history of *Sphagnum* species very complex. Moreover, the broad intercontinental geographic ranges of individual *Sphagnum* species add a layer of potential demographic and evolutionary complexity compared to most seed plant species that have much more restricted geographic ranges (Frahm & Vitt, 1993; Qian, 1999). Understanding patterns of gene flow and population size changes in closely related *Sphagnum* species is required to fully understand speciation processes and *Sphagnum* diversification.

**FIGURE 1:**
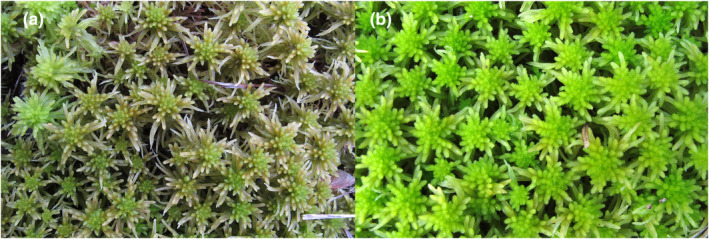
Field‐derived photographs of (a) *Sphagnum flexuosum* and (b) *Sphagnum recurvum*. Photos: Blanka Aguero (with permission).

This study focused on two closely related *Sphagnum* species: *S. recurvum* P. Beauv. and *S. flexuosum* Dozy & Molk (Figure 1). These species are members of the so‐called *S. recurvum* complex (Flatberg, 1992), which is part of the subgenus *Cuspidata*. Phylogenetic analyses (Duffy et al., 2020) have shown that *S. flexuosum* and *S. recurvum* are closely related. *Sphagnum recurvum* is restricted to eastern North America, with the exception of a single disjunct population in the Azores, while *S. flexuosum* occurs in both eastern North America and western Europe. Analyses of genetic structure have shown that European *S. flexuosum* are nested within a clade of eastern North American plants, suggesting that European plants were derived from eastern North America (Duffy et al., 2020).

The geographic distributions of *S. recurvum* and *S. flexuosum* provide a natural experiment for testing factors that impact patterns of interspecific gene flow, intraspecific gene flow between continents, and population size changes in these closely related species. Plant communities in Europe and eastern North America have been affected differently during the LGM. Europe suffered more diversity lost during the LGM (Adams & Woodward, 1989; Svenning, 2003). Fossil records have shown that there are many woody plant genera that existed in Europe during the Upper Tertiary (25–2 Mya) but now persist only in eastern North America and Asia (Adams & Woodward, 1989). One explanation for this pattern is that with the Appalachian Mountains oriented in a north–south direction, plants in eastern North America were able to freely migrate during cold periods of the Pleistocene, whereas plants in Europe were more likely blocked by the east–west orienting Alps (Hewitt, 1996; Soltis et al., 2006). Another explanation for greater diversity loss in Europe during the LGM is that southern refugia in Europe had dry climates that could not support many mesic temperate plants (Svenning, 2003). Most of the mesic temperate tree species in Europe that survived the LGM were restricted to only the Mediterranean and Black Sea regions (Svenning et al., 2008). Since *S. flexuosum* can occur only in moist habitats, the *S. flexuosum* population in Europe might have suffered a severe bottleneck or was possibly eliminated completely during the LGM, only to be reestablished by plants from eastern North America. This can result in *S. flexuosum* plants in Europe having lower genetic diversity and a smaller effective population size than plants in eastern North America. If within eastern North America, *S. flexuosum* survived glaciation south of the ice, we might predict lower genetic diversity among plants in glaciated versus unglaciated areas. On the other hand, if spore‐producing *Sphagnum* plants are highly proficient dispersers, any such genetic signal of migration and population bottlenecks could have been erased.

Opportunities for interspecific gene flow between *S. recurvum* and *S. flexuosum* were likely impacted by their intercontinental ranges. Hybridization between the species is obviously more likely between plants currently growing on the same continent, but intercontinental migration within these spore‐reproducing plants makes it possible that plants now disjunct across the Atlantic Ocean could bear signatures of gene flow as well (Shaw et al., 2014; Stenøien et al., 2011). There are several possibilities for interspecific gene flow between *S. flexuosum* and *S. recurvum*: between presently allopatric plants (i.e., eastern North American *S. recurvum* and European *S. flexuosum*), between presently sympatric plants (*S. recurvum* and eastern North American *S. flexuosum*), or between plants ancestral to current population systems (*S. recurvum* and the ancestor of both *S. flexuosum* populations).

The goals of this study were to answer the following questions. (1) are eastern North American versus European metapopulation systems within *S. flexuosum* connected by intraspecific gene flow? If so, is the rate of gene flow symmetrical between the two continents? (2) Is the rate of interspecific gene flow between *S. flexuosum* and *S. recurvum* higher between plants currently sympatric on the same continent than between plants currently separated on different continents? (3) Is there evidence of gene flow between *S. recurvum* and those ancestral to the currently disjunct populations within *S. flexuosum*? And if so, was that gene flow limited to the period during and after speciation, did it occur after secondary contact, or was it continuous? (4) Is genetic diversity in *S. flexuosum* lower in glaciated than unglaciated areas of eastern North America and Europe?

## 2 METHODS

### 2.1 Taxon sampling

**FIGURE 2:**
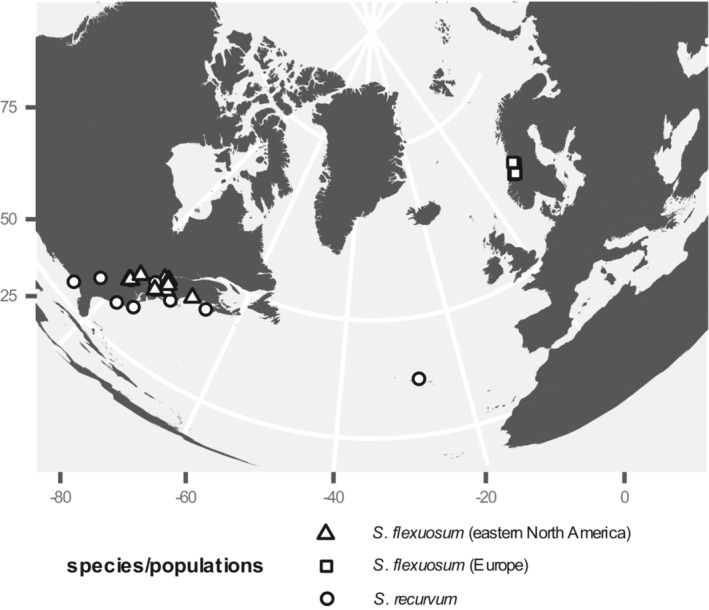
Geographic locations of *S. recurvum* and *S. flexuosum* samples used in this study.

Restriction‐site‐associated DNA sequencing (RAD‐seq) raw reads from Duffy et al. (2020) were used in this study. For DNA‐extraction, library preparation, sequencing protocols, and data availability, see Duffy et al. (2020). A total of 60 samples were divided into three groups for the present study: *S. recurvum* (16 samples), *S. flexuosum* from eastern North America (28 samples, hereafter “ENA *S. flexuosum*”), and *S. flexuosum* from Europe (16 samples, hereafter “EUR *S. flexuosum*”). All our European samples of *S. flexusosum* were collected from a relatively small area in Norway, which limits some generalities about the species in “Europe.” Recently collected samples from other areas were not available. Nevertheless, the questions we address should be relatively robust to this sampling limitation (see discussion). Figure 2 shows the geographical locations of samples used in this study. In addition to *S. flexuosum* and *S. recurvum* samples, one sample of *S. cuspidatulum* Müll. Hal. and two samples of *S. fallax* H. Klinggr. were also included for the introgression analysis. RAD‐seq reads for *S. fallax* samples were obtained from Duffy et al. (2020), while the *S. cuspidatulum* sample was acquired from *in silico* digestion of the genomic resequencing sample (see data availability for more information). Specimen voucher information is provided in the appendix (Table A1).

### 2.2 RAD‐seq data assembly

The RAD‐seq raw reads were assembled using ipyrad version 0.7.29 (Eaton, 2014) with default parameters except noted here. The reads were aligned to the *S. divinum* (v1.1) reference genome (https://phytozome‐next.jgi.doe.gov/), which is an outgroup species relative to *S. recurvum* and *S. flexuosum* (Shaw et al., 2016) in order to infer derived versus ancestral alleles. The samples were treated as haploid. Based on a previous study (Duffy et al., 2020), a read clustering threshold of 0.9 was used to maximize the number of variable sites. Loci presented in less than 80% of the samples were discarded.

### 2.3 Genetic diversity and introgression analysis

Within population nucleotide diversity (π), and pairwise F_st_, genetic distance (D_xy_) and number of fixed, shared, and monomorphic sites among ENA *S. flexuosum*, EUR *S. flexuosum*, and *S. recurvum* were calculated by the R package popgenome (Pfeifer et al., 2014). Nucleotide diversity and genetic distance are defined as the average pairwise nucleotide differences between samples within and between populations, respectively (Nei & Li, 1979). For the analysis comparing genetic diversity among geographic regions within *S. flexuosum*, two subsets of ENA *S. flexuosum* samples were created: Maryland (ten samples) and central New York (nine samples). These subsets have similar distributional ranges to the Norwegian (EUR) collections. The samples from Europe and central New York represent glaciated regions, and samples from Maryland come from an unglaciated region. Our sampling is insufficient to confirm any relationship between glacial history and genetic diversity but can yield a preliminary assessment. Nucleotide diversity was calculated for each group of *S. flexuosum* samples using the same method as above. Jackknife resampling was used to calculate the variance of nucleotide diversity estimates; *n* subsamples of each group was made by excluding one sample from the dataset, where *n* is the number samples in the group. Statistical differences of nucleotide diversity estimates were analyzed by ANOVA and post‐hoc Student's *t*‐test using Bonferroni correction for multiple comparisons. Additional samples of ENA *S. flexuosum* samples were excluded from these geographic comparisons so we could use samples from comparable areas, but these were included in other analyses.

Two ABBA/BABA site pattern statistics were calculated using the program Dsuite (Malinsky et al., 2021) to detect signatures of introgression: Patterson's *D* statistics (Green et al., 2010) and *f*_4_ ratios (Patterson et al., 2012). In the introgression analyses, outgroup samples of *S. cuspidatulum* and *S. fallax* were also included. *Sphagnum cuspidatulum* is a tropical species from Southeast Asia (Eddy, 1977). Phylogenetic analyses have shown that *S. cuspidatulum* is strongly supported as sister to *S. recurvum* (unpublished data). Including this species in the analyses allows for an inference about introgression between *S. recurvum* and the ancestor of *S. flexuosum* Europe and eastern North America. *Sphagnum fallax* was also included as an outgroup because it is one of the closest relatives to the “*S. flexuosum* + *S. recurvum”* clade (Duffy et al., 2020).

### 2.4 Demographic modeling

Multiple demographic models were compared using the approximate‐likelihood method in fastsimcoal2 (fsc26, Excoffier et al., 2013). This method uses site frequency spectra (SFS) as input. Unfolded SFS were calculated using easySFS pipeline (https://github.com/isaacovercast/easySFS). It is possible to calculate unfolded SFS in this case because the RAD‐seq reads were aligned to an outgroup reference genome, thus retaining information about derived and ancestral alleles. Since SFS requires each site to have no missing data, the easySFS pipeline allows SNPs to be subsampled from the dataset. This reduces the number of samples but increases the number of SNPs with no missing data. The populations were subsampled as follows: *S. recurvum*: 8 of 16 samples, ENA *S. flexuosum*: 16 of 28 samples, EUR *S. flexuosum*: 11 of 26 samples. Unfolded SFS were generated both using all SNPs and one SNP per RAD‐seq locus.

**FIGURE B1:**
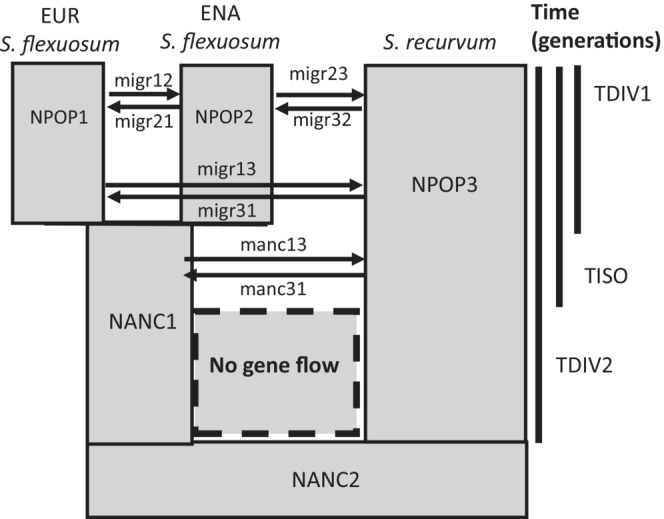
Diagram showing variable names for the demographic parameters in Figures B2, B3, B4, B5 and Tables B1

The demographic models tested all utilize the same bifurcating history: *S. recurvum* diverged from *S. flexuosum* and then *S. flexuosum* diverged into two allopatric populations; that is, Europe (EUR) and eastern North America (ENA). The differences among the models are the presence/absence of gene flow between populations. There are eight possible gene flow events: six between the three current populations and two between ancestral populations. Of the eight possible gene flow events, two are between populations of the same species (intraspecific gene flow) and six are between populations of different species (interspecific gene flow) (Figure B1). A total of 29 demographic models with several combinations of gene flow events were included in the analysis, out of 128 possible combinations (Table B1). This includes models with no gene flow, with all possible patterns of gene flow, and with only gene flow between current populations. We also tested models in which one of the eight gene flow events was excluded. Further model testing was designed by excluding multiple gene flow events that might impact the likelihood of the model when absent. All gene flows were treated as temporally continuous. After identifying the best demographic model, further comparisons were conducted by modifying the gene flow between *S. recurvum* and the ancestor of the two allopatric *S. flexuosum* populations as continuous gene flow, early gene flow shortly after divergence of the population systems, and secondary contact.

Approximate likelihoods for each demographic model were calculated by fastsimcoal2 in two steps, following Bagley et al. (2017). First, demographic parameters were inferred from the SFS containing all SNPs. According to Excoffier et al. (2013), the use of linked SNPs should not bias demographic parameter estimation and can help increase the amount of information for parameter inference. At least 100 independent runs were performed for each model. In each run, the expected SFSs were generated from 50,000 simulations, and the demographic parameters were optimized in 40 ECM cycles. In the second set of analyses, the best demographic parameters for each model were used to compute the approximate likelihood based on SFS containing only one SNP per RAD‐seq locus. In this case, the expected SFS was generated from 10 million simulations to increase the accuracy of the approximate likelihood. This approximate likelihood was then used to calculate an Akaike information criterion (AIC) for the model.

Confidence intervals of demographic parameters in the best model were obtained from parametric bootstrap. Demographic parameters in the best model were used to simulate 100 independent SFSs. For each of the simulated SFS, ten independent runs were performed using 50,000 simulations and 40 ECM cycles. Demographic parameters from the best run of each simulated SFS were then combined to calculate confidence intervals.

## 3 RESULTS

### 3.1 RAD‐seq reads assembly

The total number of raw reads from 60 samples was 90,196,383, ranging from 375,249 to 2,594,985 reads per sample (median ± SD = 1,565,588.5 ± 572,048.9). The assembly pipeline yielded 14,874 loci that are present in more than 80% of the samples, and 13,756 of those contained one or more SNPs. The mean SNP coverage was 75.12%.

### 3.2 Genetic diversity analysis

The DNA sequence matrix used in these calculations contained 282,865 sites, of which 12,307 were biallelic. *S. recurvum* has higher nucleotide diversity (π = 0.00626) than *S. flexuosum* (π = 0.00462). Both *F*_st_ and genetic distance (*D*_xy_) values between *S. recurvum* and *S. flexuosum* (*F*_st_ = 0.594, *D*_xy_ = 0.0134) are higher than the value between allopatric ENA and EUR populations of *S. flexuosum* (*F*_st_ = 0.104, *D*_xy_ = 0.00482). EUR *S. flexuosum* and *S. recurvum* share 1549 polymorphic sites and have 1403 fixed differences. ENA *S. flexuosum* and *S. recurvum* share 2061 polymorphic sites and have 1323 fixed differences. These measurements suggest that differentiation between *S. recurvum* and *S. flexuosum* is higher than that of the allopatric populations of *S. flexuosum* (Table 1).

**TABLE 1:** Nucleotide diversity within populations (π) and pairwise comparisons of the fixation index (F_st_), genetic distance (D_xy_), shared polymorphic sites, and fixed differences between *S. recurvum* and populations within *S. flexuosum*

Within population		
Population	*N*	Nucleotide diversity
*S. recurvum*	16	0.00627
ALL *S. flexuosum*	44	0.00463
EUR *S. flexuosum*	16	0.00403
ENA *S. flexuosum*	28	0.00460

Between population				
Population pair	Fst	D_xy_	Shared polymorphic sites	Fixed Differences
ALL *S. flexuosum* and *S. recurvum*	0.594	0.0134	2287	1274
EUR *S. flexuosum* and ENA *S. flexuosum*	0.104	0.00482	2904	0
EUR *S. flexuosum* and *S. recurvum*	0.615	0.0134	1549	1403
ENA *S. flexuosum* and *S. recurvum*	0.597	0.0135	2061	1323

Abbreviations: ALL, including both ENA and EUR samples; ENA, eastern North America; EUR, Europe.

Within *S. flexuosum*, nucleotide diversity in ENA *S. flexuosum* (π = 0.00460) is higher than that of EUR *S. flexuosum* (π = 0.00403) (Table 1). However, these estimates were incomparable since EUR *S. flexuosum* samples were collected from much smaller range than ENA *S. flexuosum*. Nevertheless, when ENA *S. flexuosum* was reduced into two subsets with comparable sampling range as EUR *S. flexuosum*, estimates for the three regions are significantly different (*p* < .05). Plants from Maryland (ENA, unglaciated) have the highest nucleotide diversity (π = 0.00436), followed by central New York (ENA, glaciated) (π = 0.00418), and central Norway (EUR, glaciated) has the lowest nucleotide diversity (π = 0.00403) (Table 2).

**TABLE 2:** Nucleotide diversity (π) for populations within *S. flexuosum* and statistical comparison using ANOVA and Student's *t*‐test

Summary				
Population	Continent	Glaciation during LGM	*N*	Nucleotide diversity (SD)
Maryland	ENA	No	10	0.00436 (1.2 × 10^−4^)
Upstate New York	ENA	Yes	9	0.00418 (9.7 × 10^−5^)
Central Norway	EUR	Yes	16	0.004031 (1.2 × 10^−5^)

ANOVA					
Source of variation	SS	df	MS	*F*	*p*‐value (α = 0.05)
Between groups	7.27 × 10^−7^	2	3.63 × 10^−7^	57.1	2.75 × 10^−11^
Within groups	2.03 × 10^−7^	32	6.36 × 10^−9^		
Total	9.29 × 10^−7^	34			

Post‐hoc Student's *t*‐test	
Population comparisons	*p*‐value (α = 0.0167)
Maryland – Upstate New York	0.000987
Maryland – Central Norway	3.91 × 10^−13^
Upstate New York – Central Norway	6.89 × 10^−5^

Abbreviations: ENA, eastern North America; EUR, Europe.

### 3.3 Introgession estimates: ABBA/BABA site patterns analyses

**FIGURE 3:**
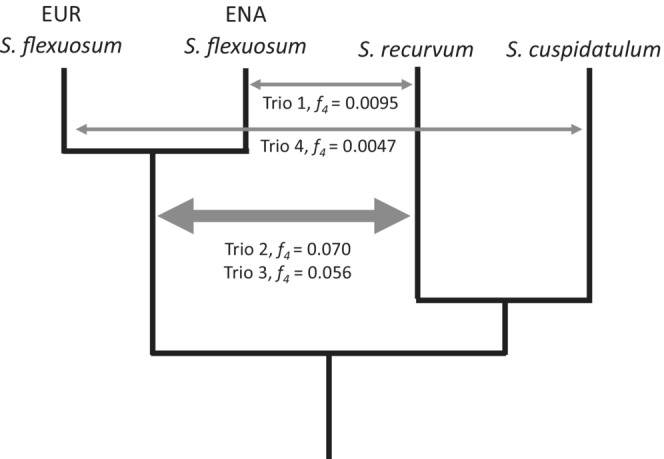
ABBA/BABA statistics analysis. The double‐headed arrows represent the putative introgression events, numbers of species trio are based on Table 3, and *f*_4_ values represent the proportion of genome involved in introgression. Thick arrow is used for introgression events with *f*_4_ higher than 0.01.

In all four species trios (Table 3), P1 and P2 share more derived alleles (BBAA sites, pattern concordant with species tree) than either P1 or P2 with P3 (ABBA/BABA sites, patterns discordant with species tree), confirming the topology of phylogenetic relationships used in this analysis (Figure 3). For the discordant site patterns, under a hypothetical scenario with only incomplete lineage sorting and the phylogeny being (([P1, P2], P3), outgroup), it is expected that P1 and P2 share equal numbers of derived alleles with P3. That is, the number of ABBA and BABA sites should be roughly equal (D‐statistics not significantly different from zero). In all four species trios, D‐statistics were significantly different from zero (*p* < .05). Table 3 and Figure 3 summarize ABBA/BABA site patterns statistics for each species trio.

**TABLE 3:** ABBA/BABA site pattern statistics. Z‐score and *p*‐value of *D* statistics were computed by jackknife. Significant *p*‐value (<.0125) suggests the presence of introgression.

no. of species trio	P1	P2	P3	*D* statistics	Z‐score	*p*‐value	f4‐ratio	BBAA sites	ABBA sites	BABA sites	Putative introgression pair
1	ENA *S. flexuosum*	EUR *S. flexuosum*	*S. cuspidatulum*	0.0534438	2.50067	.00620	0.00475	5441.56	392.791	352.936	EUR *S. flexuosum* and *S. cuspidatulum*
2	*S. cuspidatulum*	*S. recurvum*	ENA *S. flexuosum*	0.13912	5.08219	1.87 E‐07	0.0695	1900.53	1486.8	1123.63	*S. recurvum* and ENA *S. flexuosum*
3	*S. cuspidatulum*	*S. recurvum*	EUR *S. flexuosum*	0.112862	4.49452	3.49 E‐06	0.0557	1921.5	1473.49	1174.62	*S. recurvum* and EUR *S. flexuosum*
4	EUR *S. flexuosum*	ENA *S. flexuosum*	*S. recurvum*	0.0362019	2.67152	.00378	0.00949	8913.22	1203.59	1119.49	ENA *S. flexuosum* and *S. recurvum*

*Note*: *f*_4_‐ratio indicates proportion of the genome involved in introgression. In all of species trios, *S. fallax* was used as an outgroup population.

These non‐zero *D* statistics value suggest a signature of introgression, and *f*_4_‐ratio values show the proportions of genomes that were introgressed (Table 3). In the first species trio, EUR *S. flexuosum* (P1) shares more derived alleles with *S. cuspidatulum* (P3) relative to ENA *S. flexuosum* (P2), suggesting introgression between EUR *S. flexuosum* and *S. cuspidatulum*. In the second and third species trios, *S. recurvum* (P2) shares more derived alleles with both ENA and EUR *S. flexuosum* (P3), relative to *S. cuspidatulum* (P1), suggesting introgression between *S. flexuosum* and *S. recurvum*. In the fourth species trio, ENA *S. flexuosum* (P2) shares more derived alleles with *S. recurvum* (P3), relative to EUR *S. flexuosum* (P1), suggesting introgression between ENA *S. flexuosum* and *S. recurvum*.

### 3.4 Demographic history

**FIGURE 4:**
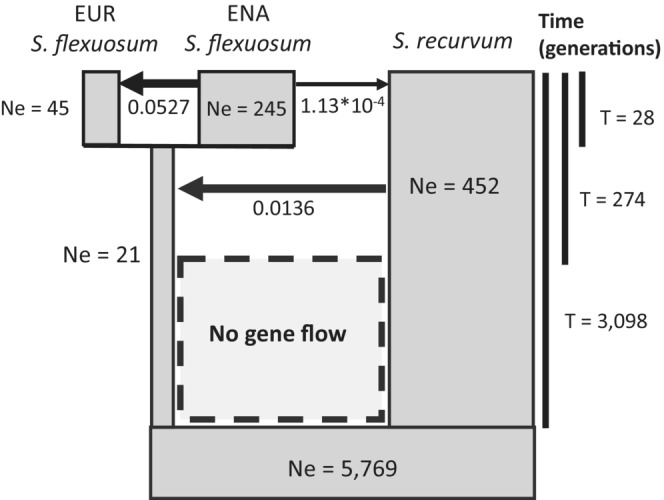
Best demographic model (“model 10 with secondary contact”). The width of the boxes is roughly proportional to the effective population sizes (N_e_); the height of the boxes is roughly proportional to divergence time; arrows represent the presence and direction of gene flow; thick arrows is used when gene flow rate exceeds 0.01.

**FIGURE 5:**
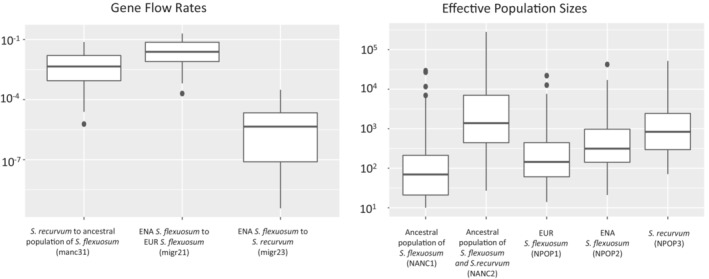
Variations in gene flow rates and effective population sizes of the best demographic model as inferred from parametric bootstrap.

**FIGURE B2:**
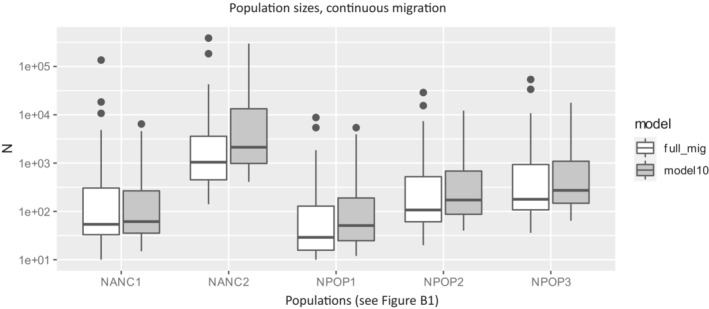
Variations in effective population sizes (*N*) inferred from parametric bootstrap of the “full migration model” and “model 10”.

**FIGURE B3:**
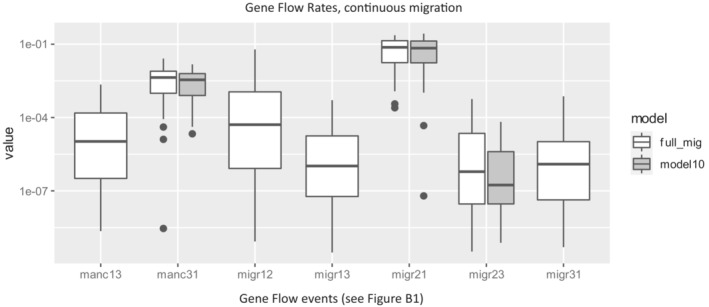
Variations in gene flow rates inferred from parametric bootstrap of the “full migration model” and “model 10”.

**FIGURE B4:**
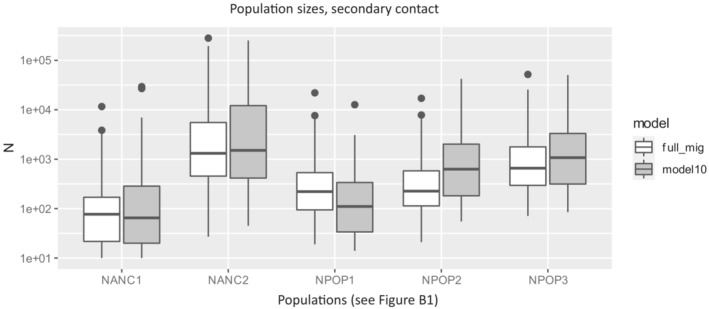
Variations in effective population sizes inferred from parametric bootstrap of the “full migration model with secondary contact” and “model 10 with secondary contact”.

**FIGURE B5:**
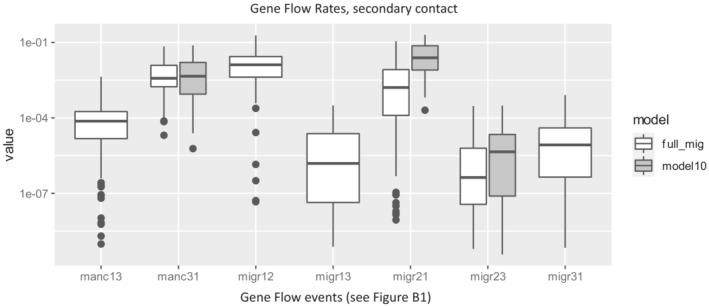
Variations in gene flow rates inferred from parametric bootstrap of the “full migration model with secondary contact” and “model 10 with secondary contact”.

Of 33 demographic models tested, the best model was “model 10 with secondary contact” (Figure 4). This model consists of three gene flow events: from ENA *S. flexuosum* to EUR *S. flexuosum*, from ENA *S. flexuosum* to *S. recurvum*, and from *S. recurvum* to the lineage ancestral to ENA and EUR *S. flexuosum*. In this model, there is also an isolation period after *S. recurvum* diverged from the ancestral population of *S. flexuosum*. For effective population sizes, EUR S. flexuosum has the smallest effective population size, followed by ENA *S. flexuosum*, and then *S. recurvum* with the largest. Figure 5 shows variation in demographic parameter estimates from parametric bootstrap. Tables B1 and B2 show demographic parameters, approximate likelihoods, and AIC values for all 33 demographic models tested. Table B2 provides 95% confidence intervals for demographic parameters in “full migration”, “full migration with secondary contact”, “model 10”, and “model 10 with secondary contact” models based on parametric bootstrap. Figures B2, B3, B4, B5 show boxplots of demographic parameters estimates for the models included in Table B2.

## 4 DISCUSSION

### 4.1 Gene flow between *S. flexuosum* and *S. recurvum*

There is much evidence that hybridization is widespread in plants and can have significant evolutionary impacts (Rieseberg, 1995; Rieseberg & Carney, 1998; Suarez‐Gonzalez et al., 2018). One possible outcome of hybridization is introgression, where hybrids backcross to one or both parental species. After generations of backcrossing, a small genomic fraction can be transferred from one species to another (Abbott et al., 2013; Edelman & Mallet, 2021). In mosses, the initial F1 hybrid is the short‐lived sporophyte generation, but meiosis in the sporangia (capsules) of such hybrids yields recombinant haploid gametophytes with allelic representation from the two parental species across loci. There is no (or little) heterozygosity to shield hybridity from natural selection. In *Sphagnum*, it is common for many species to grow intimately mixed, and demonstrably recombinant individuals have been detected in *Sphagnum* (Cronberg, 1989; Cronberg, 1998; Cronberg & Natcheva, 2002). Allopolyploid species with diploid gametophytes and tetraploid sporophytes have also been documented in *Sphagnum* from all over the world (for example, Karlin et al., 2010; Ricca & Shaw, 2010; Såstad et al., 2001), and these provide further evidence that hybridization can and does occur in the genus.

The results from both ABBA/BABA statistics and demographic modeling strongly suggest that there has been gene flow between *S. flexuosum* and *S. recurvum*. Furthermore, interspecific gene flow occurred in at least two phases: before and after the divergence between European and eastern North American plants within *S. flexuosum*. Demographic modeling indicates that the first hybridization event(s) occurred between *S. recurvum* and the ancestor of divergent North American and European *S. flexuosum* and the second event(s) between *S. recurvum* and regionally sympatric North American plants of *S. flexuosum* after the divergence of the European clade. Two phases of gene flow can also be indirectly inferred from ABBA/BABA statistics. When *S. cuspidatulum* is treated as P1 and *S. recurvum* is P2, the results indicate that *S. recurvum* shares more derived alleles with both European and eastern North American *S. flexuosum*. This pattern suggests hybridization between *S. recurvum* and the ancestor of European and eastern North American *S. flexuosum*. When European *S. flexuosum* is treated as P1, North American *S. flexuosum* as P2, and *S. recurvum* as P3 in the analyses, it is eastern North American *S. flexuosum* that shares more derived alleles with *S. recurvum*. This suggests that there was another introgression event that occurred between *S. recurvum* and *S. flexuosum* in eastern North America, but not with *S. flexuosum* in Europe. Consistent with that interpretation, *S. recurvum* shares more polymorphic sites, fewer fixed differences, and a lower estimated *F*_st_ with eastern North American *S. flexuosum* than with European *S. flexuosum*.

The best demographic model suggests that gene flow between *S. recurvum* and the ancestor of European and eastern North American *S. flexuosum* occurred during secondary contact after a period of isolation. From this, it can be inferred that speciation of *S. recurvum* and *S. flexuosum* may have occurred in allopatry. While their ranges are currently sympatric in eastern North America today, *S. flexuosum* and *S. recurvum* might have had allopatric distributions in the past. Duffy et al. (2022) showed that some continuously distributed eastern North American species of *Sphagnum* exhibit population structure that suggests regional divergence that presumably developed during previous periods of allopatry.

Based on *f*_4_ values, at least 5.5% of the genome has been transferred between *S. flexuosum* and *S. recurvum* in the first phase of gene flow. The best demographic models suggest that the direction of transfer has been from *S. recurvum* to *S. flexuosum*. This direction of gene flow is consistent across all of the demographic models tested (Table B1). The best model indicates that the population size of *S. flexuosum* after divergence from *S. recurvum* was very small (Ne = 21), relative to the ancestral population size (Ne = 5769). With this small population size, genetic drift can have an enormous influence on the gene pool; beneficial alleles might be eliminated, or slightly deleterious mutations could be fixed. Gene flow with *S. recurvum* during secondary contact could have reintroduced beneficial alleles in the ancestral population that were lost or could have introduced new alleles that originated within *S. recurvum*.

Since *S. recurvum* is restricted to eastern North America (except for one site in the Azores), this first phase of interspecific gene flow also suggests that the ancestral population of the current *S. flexuosum* populations occurred in eastern North America. This is consistent with the phylogenetic inference that *S. flexuosum* in Europe was derived from plants in eastern North America (Duffy et al., 2020).

The second phase of gene flow occurred between *S. recurvum* and eastern North American *S. flexuosum*. Both the rate of gene flow estimated by demographic modeling and the *f*_*4*_ value from ABBA/BABA statistics clearly indicate that the magnitude of interspecific gene flow before the divergence of European and North American *S. flexuosum* was higher than in the second phase after they diverged. This result suggests that reproductive isolation between sympatric *S. flexuosum* and *S. recurvum* in eastern North America is strong, even if not absolute. The relatively high estimate for interspecific gene flow before the divergence of *S. flexuosum* compared to gene flow after divergence of continental populations is consistent across most demographic models tested.

In contrast to the earlier phase of introgression between *S. recurvum* and the ancestor of North American and European *S. flexuosum*, the direction of the second phase of interspecific gene flow appears from the best demographic model to have occurred from eastern North American *S. flexuosum* into *S. recurvum*. The inferred direction of gene flow is not consistent across the models tested, but the occurrence of gene flow is strongly and consistently supported. Since this gene flow occurs only with North American *S. flexuosum*, it could contribute to the differentiation between intercontinentally disjunct populations of *S. flexuosum*.

Gene flow could potentially result in merger of two differentiated clades. However, even if hybridization is still occurring between *S. recurvum* and *S. flexuosum* in eastern North America, this appears unlikely because the rate of gene flow is low. If the value of parameters in the best demographic model are assumed to be accurate, the value of *N*_*e*_ *m* (number of individuals migrating per generation) between *S. flexuosum* and *S. recurvum* is 452*(1.13*10^−4^) = 0.051, or around 1 individual per 20 generation. Under Wright's Island model for haploid organisms, $N_{e}m=\frac{1}{2}\left(\frac{1}{F_{\mathrm{st}}}-1\right)$, in order to have an equilibrium *F*_st_ of 0.1, the value of *N*_*e*_ *m* has to be 4.5 individuals per generation (Cutter, 2019). The current rate of gene flow between *S. flexuosum* and *S. recurvum* is too low to homogenize the two species in the long run.

### 4.2 Relative genetic diversity and intercontinental gene flow in *S. flexuosum*

The last glacial maximum caused huge changes in species distributions and genetic structure of organisms in the Northern Hemisphere (Abbott & Brochmann, 2003; Hewitt, 2000). Plants in areas previously covered by ice sheets could have survived in local refugia or could have been extirpated and recolonized from another continent after the ice sheet receded. It has been shown that for angiosperms, Europe suffered more diversity losses during the last glacial maximum than did those in North America (Adams & Woodward, 1989; Svenning, 2003). With its intercontinental amphi‐Atlantic distribution, and with most of its current distribution located in areas previously glaciated, *S. flexuosum* provides an opportunity to compare genetic diversity between glaciated and unglaciated regions in Europe and eastern North America.

Due to the limited sampling range of EUR *S. flexuosum* samples, a generalized comparison of genetic diversity between *S. flexuosum* in eastern North America and Europe cannot be made with the sample we have. In order to compare samples from the two continents, we selected two subsets of ENA *S. flexuosum* samples from smaller regions: Maryland and central New York. Our estimates indicate that European plants have lower nucleotide diversity than both of the eastern North American regions. Moreover, within eastern North America, *S. flexuosum* plants from the unglaciated region (Maryland) had higher nucleotide diversity than plants from glaciated regions (central New York) (Table 2). Since all of EUR *S. flexuosum* samples were collected from central Norway, which is also glaciated, this suggests that plants in unglaciated areas have higher genetic diversity than plants in glaciated areas. Nevertheless, this observation needs to be tested with additional sampling from more glaciated and unglaciated areas.

Previous analyses of phylogenetic structure have shown that European *S. flexuosum* forms a monophyletic group that is nested within eastern North American plants (Duffy et al., 2020), suggesting that extant plants originated in North America and subsequently expanded to Europe. This inference coincides with our best demographic model, which indicates that gene flow has occurred in one direction from eastern North America to Europe. European and North American plants of *S. flexuosum* are clearly genetically similar, with *F*_st_ values much lower than interspecific *F*_st_ of either *S. flexuosum* group (European, North American) with *S. recurvum*. Moreover, there are no fixed differences between *S. flexuosum* in eastern North America versus Europe in contrast to thousands of fixed differences between *S. recurvum* and *S. flexuosum* at the species level (Table 1).

However, when considering demographic models with all possible migration events, migration rates and directions between *S. flexuosum* in Europe and eastern North America are not consistent (Tables B2 and B3). In the “full migration” model, the migration rate from eastern North America to Europe is 20 times higher than the rate from Europe to eastern North America. This pattern corresponds to the best demographic model. However, in the “full migration with secondary contact” model, the pattern is reversed; the migration rate from eastern North America to Europe is approximately half the migration rate from Europe to eastern North America. Nevertheless, the inference that *S. flexuosum* plants in Europe and eastern North America are connected by gene flow is consistent across most of the demographic models tested.

Integrating inferences about contrasting levels of genetic diversity between plants of *S. flexuosum* in North America and Europe, phylogenetic relationships among plants on the two continents, and evidence for intercontinental gene flow, we suggest the following historical scenario. *Sphagnum flexuosum* was extirpated in Europe during the LGM and was recolonized by North American plants during the Holocene. A founder effect associated with that recolonization gave rise to the lower level of genetic diversity in Europe relative to North America. An alternative scenario is that *S. flexuosum* originated in eastern North America and expanded its range to Europe, subsequent to speciation. Then, *S. flexuosum* plants in Europe have persisted through the glaciation periods in some refugia within the continent. A weak signal of introgression between European *S. flexuosum* and *S. cuspidatulum*, currently restricted to Asia, could suggest that during glaciation, *S. flexuosum* persisted in some part of Europe that is close to Asia. This is possible, although if that were the case, we might expect a stronger introgression signal between European *S. flexuosum* and *S. capidatulum* than we detected. The weak introgression signal detected here could have come from long‐distance dispersal between current populations of *S. flexuosum* in Europe and *S. cuspidatulum* in Asia. Although we cannot date the origin of *S. flexuosum* confidently because of the absence of fossils for calibration, recent estimates for the diversification of extant *Sphagnum* species suggest dates on the order of at least 10 million years ago (e.g., Shaw et al., 2010, 2019). Nevertheless, it seems unlikely that a genetic signature of any bottleneck associated with that ancient speciation process and range expansion persists today. Another important signature of recent population divergence is that there is no fixed difference between *S. flexuosum* in eastern North America and Europe. Thus, the difference in genetic diversity detected here is more likely to be caused by recent events and possibly associated with glaciation. This scenario corresponds to an earlier work by Ledent et al. (2019) which showed that post‐glacial assembly of European bryophytes involves high contribution of migrants from other continents.

Stenøien et al. (2011) inferred from microsatellite data that European plants of the the amphi‐Atlantic species *Sphagnum angermanicum* were established relatively recently from eastern North America plants via long‐distance dispersal. European plants of *S. angermanicum*, like those of *S. flexuosum*, are less genetically diverse than are those in eastern North America. In contrast, demographic analyses of other amphi‐Atlantic bryophytes have shown that levels of genetic diversity in European and North American populations are similar; bottleneck events of similar magnitudes have also been inferred on both continents (Désamoré et al., 2016). This is also the case in some circumarctic angiosperms (Brochmann & Brysting, 2008). Such demographic patterns could reflect the occurrence of northern refugia in both Europe and North America where both bryophytes and Arctic angiosperms could survive the glaciation. This discrepancy in the effects of the LGM can be explained by the difference in plant response to climate during the LGM. Paleoclimactic data have shown that ice‐free areas in Europe were drier than in eastern North America, which can produce severe effects on plants that cannot tolerate drought (Svenning, 2003). A study using species distribution modeling on European trees during the LGM has shown that boreal species have existed in northern refugia across the plains of Central and Eastern Europe, while nemoral species were restricted to southern refugia such as the Mediterranean and Black Sea regions (Svenning et al., 2008). Furthermore, a comparison of niche requirements of the relictual and extinct plant taxa in Europe has shown that relictual taxa are more cold and drought‐tolerant than the extinct taxa (Svenning, 2003). Studies of genetic diversity of bryophytes within glaciated and unglaciated areas of Europe also yielded similar patterns. Plants of the epiphytic bryophyte *Leucodon sciuroides*, which relies on host trees, have lower genetic diversity in glaciated areas than unglaciated areas (Cronberg, 2000). On the other hand, the cold‐tolerant *Hylocomium splendens* appears to have a center of genetic diversity in Northern Scandinavia, which was glaciated (Cronberg et al., 1997). Thus, since *Sphagnum* requires mesic habitats, it is reasonable to expect that *Sphagnum* in Europe would have been affected by the LGM in ways similar to temperate angiosperms that were less able to tolerate drought.

### 4.3 Limitations associated with the inference of demographic models

Our results provide some clear inferences about genetic diversity and gene flow (both intraspecific and interspecific) in *S. flexuosum* and *S. recurvum*. There are, nevertheless, important limitations and uncertainty associated with the data and approaches used in this study. With regard to sampling, all our collections of *S. flexuosum* in Europe came from central Norway (Figure 2; Table A1), even though *S. flexuosum* is widespread in Europe. Furthermore, central Norway is the northern edge of *S. flexuosum* in Europe (Laine et al., 2018) and might not represent the actual genetic diversity of *S. flexuosum* in Europe. Thus, the effective population size of EUR *S. flexuosum* as inferred from the demographic model might be lower than the actual value. Instead of using effective population size estimates from the demographic model, we reduce the scope of the question to only comparing the genetic diversity of *S. flexuosum* in glaciated areas and unglaciated areas within Europe and eastern North America. In this case, genetic diversity was used as a proxy for effective population size since the two values are correlated according to the neutral model (Ellegren & Galtier, 2016; Kimura, 1983). Variation in mutation rates can alter the relationship between genetic diversity and effective population size, but since this study focuses on plants from the same species, it can be assumed that the mutation rates are similar in all the groups being compared. There are empirical evidence showing positive correlation between genetic diversity and effective population sizes (Hague & Routman, 2016; Leimu et al., 2006).

There are also caveats regarding the interpretation of demographic models. *Sphagnum* life history does not strictly correspond to the Wright–Fisher model used in SFS simulations. The mutation rate of *Sphagnum* is unknown, and the default value of 2.8 × 10^−8^ mutations per site per generation was used in this study. Estimated values for demographic parameters should be considered relative values, not absolute values.

Moreover, in complex demographic models, different combinations of parameters can give similar approximate likelihoods. The most complex model in this study contained 19 parameters, and it can be difficult to reach the global optimum in parameter space. For some models, there can be a set of parameters that explain the data even better than the best model, but those set of parameters were not evaluated. It is also possible that 100 independent runs per model are not enough to adequately cover the parameter space. This can be problematic if there are multiple demographic models with similar approximate likelihood but have substantially different values of demographic parameters. In this case, it will be difficult to determine the best demographic model. Thus, in addition to the best demographic model reported here, it is prudent to compare parameter estimates of the best model with other models that have similar approximate likelihoods, especially the “full migration model” which contains all demographic parameters.

## 5 CONCLUSIONS

This study supports the interpretation that *S. flexuosum* in glaciated areas has lower genetic diversity than unglaciated areas, that plants in Europe are derived from eastern North America, and that the population systems disjunct across the Atlantic Ocean are still connected by gene flow. Interspecific gene flow between *S. flexuosum* and *S. recurvum* occurred in at least two phases: before and after population divergence of *S. flexuosum*. Gene flow before population divergence of *S. flexuosum* has much higher magnitude than gene flow after population divergence, and it occurred through secondary contact. Gene flow after population divergence of *S. flexuosum* occurred only between sympatric plants in eastern North America.

## AUTHOR CONTRIBUTIONS

**Karn Imwattana:** Conceptualization (equal); formal analysis (lead); investigation (lead); writing – original draft (lead); writing – review and editing (equal). **Aaron Duffy:** Data curation (supporting). **Blanka Aguero:** Data curation (supporting). **Jon Shaw:** Conceptualization (supporting); supervision (lead); writing – review and editing (equal).

## Data Availability

*In silico* digested reads from a genomic resequencing sample of *S. cuspidatulum* (Library IUSS) are available in Dryad (https://doi.org/10.5061/dryad.1c59zw3xc). Demultiplexed Illumina reads from RAD‐seq samples of *S. flexuosum*, *S. recurvum*, and *S. fallax* are available in Dryad https://doi.org/10.5061/dryad.1g1jwsts7 (Duffy et al., 2020). For the list of samples used in this study, see Appendix A.

## APPENDIX A

**TABLE A1:** Voucher information of the specimens used in this study, for more information see Duffy et al. (2020).

DNA isolate	Species	Geographic region	Collectors	Col. nr.	Country	State/province	County/district	Collection date	Latitude	Longtitude
SB4974	*S. flexuosum*	Eastern North America	A. Garrett	A030	USA	West Virginia	Grant Co.	20‐Jun‐13	39.45417	−79.38663
SB4975	*S. flexuosum*	Eastern North America	A. Garrett	A031	USA	West Virginia	Grant Co.	20‐Jun‐13	39.45417	−79.38663
SB4976	*S. flexuosum*	Eastern North America	A. Garrett	A033	USA	Maryland	Garrett Co.	20‐Jun‐13	39.56775	−79.29842
SB4980	*S. flexuosum*	Eastern North America	A. Garrett	A043	USA	Maryland	Garrett Co.	21‐Jun‐13	39.56448	−79.23805
SB4985	*S. flexuosum*	Eastern North America	B. Shaw	18,906	USA	Maryland	Garrett Co.	21‐Jun‐13	39.5645	−79.2382
SB4987	*S. flexuosum*	Eastern North America	B. Shaw	1107a/1	USA	Maryland	Garrett Co.	21‐Jun‐13	39.66966	−79.08571
SB4996	*S. flexuosum*	Eastern North America	A. Garrett	A065	USA	Maryland	Garrett Co.	21‐Jun‐13	39.66565	−79.08935
SB4997	*S. flexuosum*	Eastern North America	A. Garrett	A066	USA	Maryland	Garrett Co.	21‐Jun‐13	39.66565	−79.08935
SB5002	*S. flexuosum*	Eastern North America	B. Shaw	18927	USA	Pennsylvania	Elk Co.	22‐Jun‐13	41.6802	−78.5248
SB5003	*S. flexuosum*	Eastern North America	B. Shaw	18930	USA	Pennsylvania	Elk Co.	22‐Jun‐13	41.6802	−78.5248
SB5007	*S. flexuosum*	Eastern North America	B. Shaw	18937	USA	Pennsylvania	Elk Co.	22‐Jun‐13	41.6808	−78.5253
SB5009	*S. flexuosum*	Eastern North America	A. Garrett	A075	USA	Pennsylvania	McKean Co	22‐Jun‐13	41.68032	−78.52475
SB5011	*S. flexuosum*	Eastern North America	A. Garrett	A083	USA	Pennsylvania	McKean Co	22‐Jun‐13	41.68055	−78.525
SB5019	*S. flexuosum*	Eastern North America	A. Garrett	A100	USA	New York	St. Lawrence Co.	23‐Jun‐13	44.15123	−74.9561
SB5022	*S. flexuosum*	Eastern North America	B. Shaw	18953	USA	New York	St. Lawrence Co.	23‐Jun‐13	44.1511	−74.9562
SB5023	*S. flexuosum*	Eastern North America	B. Shaw	18957	USA	New York	St. Lawrence Co.	23‐Jun‐13	44.1511	−74.9562
SB5024	*S. flexuosum*	Eastern North America	B. Shaw	18958	USA	New York	St. Lawrence Co.	23‐Jun‐13	44.1511	−74.9562
SB5025	*S. flexuosum*	Eastern North America	B. Shaw	18959	USA	New York	St. Lawrence Co.	23‐Jun‐13	44.1511	−74.9562
SB5039	*S. flexuosum*	Eastern North America	B. Shaw	18974	USA	New York	Franklin Co	23‐Jun‐13	44.4262	−74.245
SB5042	*S. flexuosum*	Eastern North America	M. Reeve	660	USA	ME	Waldo Co.	10‐Nov‐10	44.41333	−69.01833
SB5043	*S. flexuosum*	Eastern North America	J. Shaw	16491	USA	Maryland	Garrett Co.	20‐Jun‐13	39.5975	−79.27472
SB5091	*S. flexuosum*	Eastern North America	B. Shaw	18992	USA	New York	Rensselaer Co.	24‐Jun‐13	42.6449	−73.4094
SB5106	*S. flexuosum*	Eastern North America	B. Shaw	18,935	USA	Pennsylvania	McKean Co	22‐Jun‐13	41.6808	−78.5253
SB5108	*S. flexuosum*	Eastern North America	A. Garrett	A074	USA	Pennsylvania	McKean Co	22‐Jun‐13	41.68032	−78.52475
SB5120	*S. flexuosum*	Eastern North America	A. Garrett	A126	USA	New York	Warren Co.	24‐Jun‐13	43.67917	−73.78852
SB5121	*S. flexuosum*	Eastern North America	A. Garrett	A127	USA	New York	Warren Co.	24‐Jun‐13	43.67892	−73.78842
AG198	*S. flexuosum*	Europe	A. Garrett	A161	Norway	Sør‐Trøndelag	Trondheim	12‐Aug‐13	63.4224	10.26592
AG201	*S. flexuosum*	Europe	A. Garrett	A166	Norway	Sør‐Trøndelag	Trondheim	12‐Aug‐13	63.42235	10.26598
AG209	*S. flexuosum*	Europe	A. Garrett	A178	Norway	Sør‐Trøndelag	Orkdal	13‐Aug‐13	63.36198	9.85645
AG231	*S. flexuosum*	Europe	A. Garrett	A214	Norway	Sør‐Trøndelag	Melhus	14‐Aug‐13	63.30583	10.38778
AG234	*S. flexuosum*	Europe	A. Garrett	A219	Norway	Sør‐Trøndelag	Melhus	14‐Aug‐13	63.3043	10.38412
AG239	*S. flexuosum*	Europe	A. Garrett	A227	Norway	Sør‐Trøndelag	Klæbū	14‐Aug‐13	63.30897	10.39018
AG247	*S. flexuosum*	Europe	A. Garrett	A241	Norway	Nord‐Trøndelag	Steinkjer	15‐Aug‐13	63.93202	11.692
AG257	*S. flexuosum*	Europe	A. Garrett	A258	Norway	Nord‐Trøndelag	Grong	15‐Aug‐13	64.3565	12.33225
AG259	*S. flexuosum*	Europe	A. Garrett	A260	Norway	Nord‐Trøndelag	Grong	15‐Aug‐13	64.3565	12.33225
AG263	*S. flexuosum*	Europe	A. Garrett	A264	Norway	Nord‐Trøndelag	Grong	15‐Aug‐13	64.3567	12.33262
AG268	*S. flexuosum*	Europe	A. Garrett	A274	Norway	Nord‐Trøndelag	Høylandet	16‐Aug‐13	64.66405	12.17537
AG271	*S. flexuosum*	Europe	A. Garrett	A280	Norway	Nord‐Trøndelag	Høylandet	16‐Aug‐13	64.65497	12.17522
AG273	*S. flexuosum*	Europe	A. Garrett	A282	Norway	Nord‐Trøndelag	Høylandet	16‐Aug‐13	64.65493	12.17517
AG281	*S. flexuosum*	Europe	A. Garrett	A291	Norway	Sor‐Trøndelag	Klæbū	17‐Aug‐13	63.2495	10.45597
AG395	*S. flexuosum*	Europe	A. Garrett	A278	Norway	Nord‐Trøndelag	Høylandet	16‐Aug‐13	64.6577	12.18405
SB5361	*S. flexuosum*	Europe	A. Garrett	A282	Norway	Nord‐Trøndelag	Høylandet	16‐Aug‐13	63.30897	10.39018
SB4977	*S. flexuosum*	Eastern North America	A. Garrett	A037	USA	Maryland	Garrett Co.	21‐Jun‐13	39.56513	−79.23867
SB5241	*S. flexuosum*	Eastern North America	A. Garrett	A146	USA	Pennsylvania	Pike Co.	25‐Jun‐13	41.3794	−75.0792
SB4982	*S. recurvum*	Eastern North America	A. Garrett	A045	USA	Maryland	Garrett Co.	21‐Jun‐13	39.56362	−79.23695
SB4983	*S. recurvum*	Eastern North America	B. Shaw	18904	USA	Maryland	Garrett Co.	21‐Jun‐13	39.5645	−79.2382
SB4986	*S. recurvum*	Eastern North America	B. Shaw	18907	USA	Maryland	Garrett Co.	21‐Jun‐13	39.5645	−79.2382
SB4989	*S. recurvum*	Eastern North America	B. Shaw	18916	USA	Maryland	Garrett Co.	21‐Jun‐13	39.6669	−79.0873
SB4995	*S. recurvum*	Eastern North America	A. Garrett	A064	USA	Maryland	Garrett Co.	21‐Jun‐13	39.66565	−79.08935
SB5016	*S. recurvum*	Eastern North America	B. Shaw	18,945	USA	New York	Chenango Co.	22‐Jun‐13	42.496	−75.8268
SB5045	*S. recurvum*	Eastern North America	J. Atwood	8	USA	Connecticut	New London Co.	28‐Jul‐02	41.59333	−71.87
SB5048	*S. recurvum*	Eastern North America	T. Neily	766	Canada	Nova Scotia	Yarmouth Co.	22‐Aug‐12	43.91326	−65.88168
SB5109	*S. recurvum*	Eastern North America	A. Garrett	A142	USA	Pennsylvania	Pike Co.	25‐Jun‐13	41.3794	−75.0792
SB5113	*S. recurvum*	Eastern North America	B. Shaw	18,941	USA	New York	Chenango Co.	22‐Jun‐13	42.4966	−75.8275
SB5136	*S. recurvum*	Eastern North America	B. Shaw	17,793	USA	North Carolina	Dare Co.	9‐Dec‐13	35.80115	−75.88329
SB5140	*S. recurvum*	Eastern North America	Ferreira M.	1	Portugal	Azores	Terceira	4‐Jul‐08	38.73	−27.27
SB5196	*S. recurvum*	Eastern North America	B. Aguero	19,470	USA	North Carolina	Dare Co.	17‐Apr‐18	35.80102	−75.88355
SB5231	*S. recurvum*	Eastern North America	B. Piatkowski	2018‐40	USA	Florida	Franklin	17‐May‐18	30.00172	−85.00005
SB5233	*S. recurvum*	Eastern North America	B. Aguero	M02A/2	USA	North Carolina	Transylvania Co.	01‐May‐18	35.35137	−82.77266
SB5234	*S. recurvum*	Eastern North America	B. Aguero	19,605	USA	North Carolina	Brunswick Co.	04‐Jun‐18	34.09144	−75.88355
AG255	*S. fallax*	Europe	A. Garrett	A254	Norway	Nord‐Trøndelag	Sltaeginkjer	15‐Aug‐13	63.93458	11.69667
SB5032	*S. fallax*	Eastern North America	B. Aguero	18,965	USA	New York	Franklin Co.	23‐Jun‐13	44.4258	−74.2436
IUSS	*S. cuspidatulum*	Asia	K. Imwattana	KI‐149	Thailand	Chiang Mai	Chiang Mai	4‐Jul‐18	18.58885	98.48516

## APPENDIX B

Demographic models tested in this study.

**TABLE B1:** List of all tested demographic models and the inferred parameters, see Figure B1 for the definition of parameter names.

Model name	NPOP1	NPOP2	NPOP3	NANC1	NANC2	TDIV1	TDIV2	TSIO	migr12	migr13	migr21	migr23	migr31	migr32	manc13	manc31	k	Lhood	AIC
**Full_migration**	**141**	**478**	**893**	**240**	**6554**	**208**	**3139**		**7.55 E‐04**	**2.85 E‐07**	**0.0151132**	**8.56 E‐07**	**7.17 E‐06**	**9.57 E‐05**	**6.72 E‐05**	**0.0010497**	**17**	**−14700.7**	**67733.2**
no_migration	1562	3894	3842	3095	19,950	239	3666										9	−14831.0	68317.4
recent_migration	259	295	681	2884	2625	545	743		0.012888	1.72 E‐07	0.0021118	3.97 E‐05	1.91 E‐04	1.03 E‐06			15	−14782.2	68104.5
nomig12	106	400	763	255	3749	223	2254			1.51 E‐08	0.0238204	4.15 E‐06	4.97 E‐05	1.48 E‐04	8.72 E‐05	0.0010908	16	−14694.1	67700.7
nomig13	2947	6594	13,519	2825	98,886	2228	37,768		2.04 E‐04		5.99 E‐04	1.33 E‐08	4.31 E‐08	1.61 E‐07	1.88 E‐08	8.73 E‐05	16	−14691.5	67688.9
nomig21	925	499	1871	403	10,640	461	4719		0.0077547	3.42 E‐09		4.07 E‐07	4.46 E‐05	1.27 E‐05	3.58 E‐09	6.83 E‐04	16	−14721.8	67828.2
nomig23	1736	1256	4526	1124	30,897	853	12,209		0.0030612	1.54 E‐07	2.59 E‐05		8.00 E‐06	1.32 E‐06	1.48 E‐08	2.64 E‐04	16	−14700.1	67728.5
nomig31	23	84	176	44	795	38	400		1.38 E‐05	2.55 E‐05	0.1347102	9.47 E‐05		5.61 E‐04	4.93 E‐06	0.0062277	16	−14708.8	67768.6
nomig32	154	110	346	76	5213	54	1348		0.0430574	1.11 E‐07	4.41 E‐05	5.68 E‐05	2.30 E‐07		4.41 E‐06	0.0034757	16	−14710.9	67778.2
nomig_a13	36	203	275	53	6054	65	1053		2.12 E‐05	1.85 E‐07	0.0762748	1.33 E‐05	1.84 E‐07	4.33 E‐05		0.0051892	16	−14696.4	67711.4
nomig_a31	598	791	1431	3070	20,615	1445	8005		0.0057039	9.35 E‐09	0.0013189	4.12 E‐08	7.70 E‐05	2.33 E‐05	0.0032208		16	−14752.0	67967.4
model1	41	163	241	46	2799	41	782				0.0664278	1.26 E‐05		2.78 E‐05	1.40 E‐07	0.0054293	14	−14713.7	67786.9
model2	34	151	261	67	1587	83	598				0.0857	5.35 E‐05	4.53 E‐07	3.13 E‐04	7.70 E‐08	0.0046777	15	−14687.0	67666.0
model3	45	183	367	122	4215	53	2093			2.43 E‐05	0.057202	8.75 E‐08		1.44 E‐04	2.27 E‐04	0.0017993	15	−14703.8	67743.5
model4	112	453	782	157	46,458	200	3726		5.16 E‐04		0.0303858	5.39 E‐07		3.96 E‐05	7.78 E‐08	0.0020168	15	−14712.0	67781.4
model5	36	137	225	51	1369	54	528				0.0684546		1.97 E‐08	2.48 E‐04		0.0053256	13	−14692.0	67685.4
model6	51	213	312	73	1468	54	652				0.0569785		2.89 E‐05	1.30 E‐04	1.18 E‐07	0.003058	14	−14691.7	67685.7
model7	211	1060	1598	331	11,533	208	4697				0.0134329	2.36 E‐08	7.47 E‐08	6.24 E‐07		6.92 E‐04	14	−14697.0	67710.3
model8	919	3266	5489	1240	36,042	2177	14,000			1.46 E‐06	0.0029642		7.92 E‐09	1.83 E‐05		2.61 E‐04	14	−14696.4	67707.4
model9	96	289	585	122	3830	91	1653		2.69 E‐07		0.0340505		1.90 E‐06	6.94 E‐05		0.0019851	14	−14697.4	67712.2
**model10**	**49**	**170**	**307**	**58**	**9279**	**42**	**1416**				**0.0595581**	**1.52 E‐05**				**0.0040193**	**12**	**−14681.4**	**67634.4**
model11	53	171	278	63	1215	29	705				0.0447198	2.17 E‐06			2.44 E‐07	0.0032825	13	−14699.5	67719.8
model12	701	3312	5080	979	38,440	1542	12,062				0.0040156	1.22 E‐09		1.37 E‐05		2.80 E‐04	13	−14694.4	67696.0
model13	105	370	634	140	3520	88	1516				0.021999	3.67 E‐06	4.07 E‐09			0.0016853	13	−14701.0	67726.4
model14	3645	16,267	26,557	6074	145,983	4308	61,643			2.50 E‐06	7.27 E‐04	7.05 E‐08				3.85 E‐05	13	−14698.0	67712.7
model15	60	40	109	20	1481	24	360		0.0857143		1.83 E‐08	3.66 E‐08				0.0129705	13	−14714.4	67788.1
model16	16	82	134	28	600	17	269				0.1444892					0.0078581	11	−14714.5	67784.8
model17	1611	7187	8715	114,779	43,420	8624	9977				0.0014391	8.21 E‐06					12	−14836.5	68348.7
model18	15,596	29,750	46,581	31,654	174,203	2047	53,352					1.39 E‐06				3.03 E‐06	11	−14764.5	68015.2
early_gene_flow	196	634	871	290	3095	58	1766	143	3.16 E‐06	4.40 E‐08	0.0126008	8.56 E‐06	8.29 E‐05	9.51 E‐05	2.09 E‐08	9.65 E‐04	18	−14694.7	67707.5
**secondary_contact**	**629**	**802**	**2306**	**68**	**177**	**441**	**27,447**	**871**	**0.003833**	**4.67 E‐06**	**0.002031**	**7.05 E‐08**	**3.70 E‐05**	**1.01 E‐06**	**9.66 E‐05**	**0.0042645**	**18**	**−14681.2**	**67645.6**
model10_with_early_gene_flow	2948	12,332	16,923	4239	109,882	2090	42,452	2730			8.53 E‐04	9.64 E‐07				6.89 E‐05	13	−14715.0	67791.2
**model10_with_secondary_contact**	**45**	**245**	**452**	**21**	**5769**	**80**	**3098**	**274**			**0.052726**	**1.13 E‐04**				**0.013594**	**13**	**−14659.2**	**67533.9**

*Note*: Highlighted models were selected for parametric bootstrap (see Table B2; Figures B2, B3, B4, B5)

**TABLE B2:** Demographic parameters and confidence intervals of the full migration model and model 10 (the best demographic model) with continuous ancestral migration and secondary contact, see Figure B1 for the definition of parameter names.

RUN	NPOP1	NPOP2	NPOP3	NANC1	NANC2	TDIV1	TDIV2	TISO	migr12	migr13	migr21	migr23	migr31	migr32	manc13	manc31
Full migration																
Original_run	141	478	893	240	6554	208	3139		7.55 E‐04	2.85 E‐07	0.015113	8.56 E‐07	7.17 E‐06	9.57 E‐05	6.72 E‐05	0.00105
mean	301.79	979.99	1793.61	1962.77	9785.92	733.94	3615.44		0.005058	4.57 E‐05	0.086108	3.82 E‐05	3.01 E‐05	0.000527	0.00014	0.006146
median	29	107	178	54	1042.5	37	555		5.11 E‐05	1.05 E‐06	0.074566	6.11 E‐07	1.24 E‐06	0.000293	1.06 E‐05	0.004333
SD	1054.163	3362.891	6434.168	13664.72	42747.65	4557.508	11570.83		0.012711	9.89 E‐05	0.071233	9.02 E‐05	0.000101	0.000596	0.000308	0.006414
upper 95% CI	508.406	1639.117	3054.707	4641.056	18164.46	1627.212	5883.322		0.007549	6.51 E‐05	0.100069	5.59 E‐05	4.99 E‐05	0.000644	0.0002	0.007403
lower 95% CI	202.1424	658.7231	1194.887	1053.123	6225.686	415.0065	2462.309		0.003578	3.29 E‐05	0.066494	2.72 E‐05	2.04 E‐05	0.000401	0.000101	0.004695
Model 10																
Original_run	49	170	307	58	9279	42	1416				0.059558	1.52 E‐05				0.004019
mean	345.88	1029.2	1674.24	467.51	15536.13	197.48	4892.03				0.085014	5.35 E‐06				0.00432
median	51	172	272.5	61.5	2129.5	34	915.5				0.068852	1.74 E‐07				0.003497
SD	784.8463	2060.191	3304.299	1016.397	42606.33	410.0477	10308.92				0.077057	1.2 E‐05				0.004
upper 95% CI	499.7099	1432.997	2321.883	666.7237	23886.97	277.8493	6912.579				0.100117	7.71 E‐06				0.005104
lower 95% CI	247.9369	748.3325	1219.151	336.8322	10854.28	143.0215	3537.165				0.065391	3.84 E‐06				0.00332
Full migration with secondary contact																
Original_run	629	802	2306	68	177	441	27,447	871	0.003833	4.67 E‐06	0.002031	7.05 E‐08	3.7 E‐05	1.01 E‐06	9.66 E‐05	0.004265
mean	859.46	806.22	2526.23	356.77	13940.39	378.04	15306.57	3087.61	0.022695	2.52 E‐05	0.010984	1.46 E‐05	6.03 E‐05	8.09 E‐06	0.00022	0.007995
median	221.5	226	658	77	1315.5	87.5	6668	717	0.01289	1.56 E‐06	0.001609	4.29 E‐07	8.47 E‐06	1.94 E‐07	7.41 E‐05	0.003757
SD	2513.873	2047.719	6397.056	1285.493	42266.01	843.9441	21051.35	9919.357	0.029148	5.24 E‐05	0.021975	4.12 E‐05	0.000137	2.62 E‐05	0.000577	0.010577
upper 95% CI	1352.179	1207.573	3780.053	608.7265	22224.53	543.453	19432.63	5031.804	0.028408	3.54 E‐05	0.015291	2.26 E‐05	8.72 E‐05	1.32 E‐05	0.000333	0.010068
lower 95% CI	594.4329	569.5357	1785.34	237.4596	9584.383	271.5232	11497.77	2101.376	0.017127	1.82 E‐05	0.007987	1.01 E‐05	4.32 E‐05	5.5 E‐06	0.000155	0.006022
Model 10 with secondary contact																
Original_run	45	245	452	21	5769	80	3098	274			0.052726	0.000113				0.013594
mean	405.74	1920.28	3083.07	978.77	17966.4	554.84	25922.16	3334.15			0.045681	2.08 E‐05				0.00962
median	110.5	628	1076	65	1511.5	172	12653.5	755			0.024446	4.49 E‐06				0.004538
SD	1338.282	4759.928	6134.585	4075.481	39986.49	946.531	32264.66	7749.457			0.047288	4.59 E‐05				0.011854
upper 95% CI	668.0434	2853.226	4285.449	1777.564	25803.75	740.3601	32246.03	4853.044			0.05495	2.98 E‐05				0.011943
lower 95% CI	274.8035	1361.048	2243.122	630.3674	12908.86	409.7294	19601.94	2382.953			0.034911	1.49 E‐05				0.007279
